# Discrepancy in the Responsiveness to Hip Range of Motion Between Harris and Oxford Hip Scores

**DOI:** 10.1016/j.artd.2021.10.008

**Published:** 2022-01-20

**Authors:** Toshiyuki Kawai, Koji Goto, Yutaka Kuroda, Yaichiro Okuzu, Shuichi Matsuda

**Affiliations:** Department of Orthopedic Surgery, Kyoto University Graduate School of Medicine, Kyoto, Japan

**Keywords:** Total hip arthroplasty, Range of motion, Leg lengthening, Leg length discrepancy

## Abstract

**Background:**

The primary objectives of total hip arthroplasty (THA) include mobility improvement and pain relief; however, the correlation between hip range of motion (ROM) and function remains unclear. We aimed to explore how ROM affects hip functions after THA and compare the responsiveness of each component of the modified Harris Hip Score (mHHS) and Oxford Hip Score (OHS) to preoperative and postoperative ROM.

**Methods:**

This prospective observational study involved 120 patients who underwent unilateral THA. Univariate regression analyses were performed using the University of California Los Angeles activity score and mHHS and OHS to determine the effects of preoperative and postoperative flex ROM on clinical scores at 12 months. Multivariate regressions were performed to adjust for the confounding effects of patient factors: age, sex, body mass index, and diagnosis.

**Results:**

A larger preoperative flexion ROM was associated with a higher score in the mHHS socks component (standardized coefficient [SC] = 0.26, *P* = .0041) at 12 months; the effect on the OHS socks component was not significant (*P* = .34). A larger flexion ROM at 12 months was associated with higher scores in the mHHS support (SC = 0.21, *P* = .026), stairs (SC = 0.35, *P* = .0002), and socks (SC = 0.32, *P* = .0007) components but had no significant effect on any OHS component. The effects of ROM on University of California Los Angeles activity score were limited.

**Conclusions:**

A discrepancy was noted in the responsiveness to ROM between the two major measurement tools; this difference might be because mHHS and OHS are surgeon- and patient-administered questionnaires, respectively. This discrepancy also suggests that the patients have higher satisfaction than that assumed by the surgeons.

## Introduction

Pain and limited mobility are common symptoms of hip osteoarthritis (OA). Restricted hip range of motion (ROM) increases lower limb disability in patients with hip OA [1,2]. Thus, the primary objectives of total hip arthroplasty (THA) are mobility improvement and pain relief [3].

Limited studies have been conducted regarding the correlation between hip ROM and function score. Therefore, the correlation between hip ROM and function remains controversial; some studies have reported ROM as a determinate of function [2,4], whereas others have reported poor correlations [5,6]. Previous studies have primarily reported the effects of preoperative ROM on preoperative function and that of postoperative ROM on postoperative function. To our knowledge, there is no study on the effects of preoperative ROM on postoperative function. A previous study has reported that a higher flexion ROM after THA is associated with a higher postoperative Harris Hip Score (HHS) [7]. Another study found that patients with restricted preoperative hip joint flexion (<95°) had greater activity limitations before surgery than patients with nonrestricted flexion (>95°) [8]; however, a poor correlation existed between ROM in patients with hip OA and Western Ontario and McMaster Universities Osteoarthritis Index Pain or Function Scale [8].

Hip ROM can have different effects on various clinical outcome measurement systems; however, these also remain unknown. The HHS and Oxford Hip Score (OHS) are the two most frequently used clinical measurement tools. The HHS is a surgeon-administered joint-specific questionnaire comprising 10 items: 2 (ROM and absence of deformity) for the physician physical examination component and 8 for the patient-reported outcome measure (PROM) component. When only the PROM component of the HHS questionnaire is used, it is referred to as modified HHS (mHHS). The OHS is a 12-item self-administered questionnaire. These two measurement tools share similar questions regarding function components (limp, stairs, and socks) and are considered to be strongly correlated [9,10]. Correlations between the components of these two tools were examined, and significant correlations were found in pain, walking, limp, socks, and stairs components [9]. However, the difference in sensitivity of each tool to hip ROM is yet to be examined.

This study aimed to explore how the preoperative ROM affects postoperative hip functions after THA and compare the responsiveness of each component of mHHS and OHS to preoperative and postoperative ROM.

## Material and methods

This was a prospective observational study. All patients provided informed consent, and the study protocol was approved by the institutional review board of our hospital.

Between July 2014 and July 2016, 200 primary THAs (in 183 patients) were performed at our institute. Among these, 78 THAs were excluded because the condition of the contralateral hip might have affected the clinical activity scores. Specifically, the contralateral hips of the excluded patients had untreated severe OA (n = 39), a total hip joint implanted within 12 months before the index surgery (n = 24), and untreated osteonecrosis of the femoral head (n = 15). Two more THAs were excluded because the procedure involved subtrochanteric shortening osteotomy. Therefore, the present study cohort included 120 THAs in 120 patients whose contralateral hip was either normal or had undergone THA within the prior year.

This cohort was the same group of patients used in another prospective study that aimed to evaluate the changes in the PROM after THA [11].

All 120 procedures were performed by one of the senior hip surgeons (K.G., Y.K., or K.S.). All surgeries were performed via the anterolateral approach [12]. A cemented stem was used in 89 hips, whereas an uncemented stem was used in 31 hips. On the acetabular side, a cemented cup was used in five hips, whereas an uncemented cup was used in 115 hips. The implant choice was made on the basis of the surgeon’s preference.

The following patient-related covariates were considered: age, sex, body mass index (BMI), and diagnosis.

### Clinical scores

Clinical scores were evaluated preoperatively and postoperatively at 3, 6, and 12 months using the mHHS, University of California Los Angeles activity score (UCLAAS), and OHS. All patients were evaluated using all three scores. The standard HHS comprises 10 questions: two (ROM and absence of contracture) for the physical examination component and eight for the PROM component. In this study, the mHHS comprised only the eight PROM components but not the ROM and contracture components included in the standard HHS.

The OHS was calculated in accordance with the modified scoring system; each question was scored from 0 to 4, with 4 representing the best outcome or the least symptoms. The OHS ranged from 0 to 48, with 48 indicating the best outcome. One of the surgeons filled out the mHHS questionnaire by asking each question to the patients; the UCLAAS questionnaire was also filled out by a surgeon in a similar manner. The OHS questionnaire was handed out to the patients; it was collected by the hospital staff after the patients had completed the questionnaire.

The hip flexion ROM was measured preoperatively and postoperatively at 3, 6, and 12 months after THA by one of the senior orthopedic surgeons through visual estimation. Previous studies have revealed that the intraclass correlation coefficients (ICCs) for the interobserver reliability and intraobserver reproducibility of hip ROM measurements obtained through visual estimation are the highest for hip flexion [13,14]. In the present study, hip flexion was used as a representative direction of movement because of its high ICCs for interobserver and intraobserver measurement reliabilities compared with the hip ROM in other directions. Patients were divided into two groups according to the preoperative flex ROM: small (preoperative flex ROM < 90°) and large (preoperative flex ROM ≥ 90°) preoperative flex ROM groups. Similarly, patients were also divided into small and large postoperative ROM groups, with a flex ROM of 90° at 12 months as the threshold.

### Statistical analysis

Differences in proportions were analyzed using the chi-square test. Differences in means were analyzed using the Wilcoxon test for the comparison of two groups. Univariate regression analyses were performed to determine the effects of preoperative and postoperative flex ROM on clinical scores (mHHS, OHS, and UCLAAS) at 12 months. Multivariate regressions were performed to adjust the confounding effects of patient factors: age, sex, BMI, and diagnosis (OA or others). Probability values of <0.05 were considered significant. The sample size required for between-group comparison was calculated using bilateral alpha risk of 5%, 90% power, standard deviation, and anticipated differences for each function score. At least 97 patients were required. With an anticipated exclusion rate of 40%, we included 183 patients for analyses. All statistical analyses were performed using the JMP Pro 14 software (SAS Institute, Cary, NC). The correlations between clinical measurement scores (mHHS, OHS, and UCLAAS) and ROM at each time point were also visualized using the nonparametric Kernel density estimation using a standard deviation multiplied by 0.225 (the default setting for JMP Pro 14) for each variable as the Kernel standard deviation for each variable. Quantile density contours were drawn at every 0.05.

## Results

Patient demographics are presented in Table 1. There were no significant differences in sex, age, BMI, and diagnosis between the small and large ROM groups. The large ROM group had more women, but the difference was not statistically significant (*P* = .073).

**Table 1:** Patient demographics.

Variable		All cases (n = 120)	Preoperative flexion ROM < 90 (n = 59)	Preoperative flexion ROM = 90 or larger (n = 61)	*P* value
Sex (female)		90 (75.0%)	40(67.8%)	50(82.0%)	.073
Age		62.4 ± 11.9	61.1 ± 11.2	63.7 ± 12.5	.15
BMI		23.7 ± 3.8	24.2 ± 3.9	23.2 ± 3.6	.13
Diagnosis	OA	94 (78.3%)	44 (74.6%)	50 (82.0%)	.58
	ONFH	22 (18.3%)	13 (22.0%)	9 (14.8%)	
	RDC	4 (3.3%)	2 (3.4%)	2 (3.3%)	
Preoperative flex ROM		80.9 ± 20.9	65.4 ± 18.8	95.9 ± 8.0	<.0001
Flex ROM at 12 mo		95.0 ± 13.3	89.5 ± 14.9	100.3 ± 8.9	<.0001

Values are expressed as mean (percent) or mean ± standard deviation.

ONFH, osteonecrosis of the femoral head; RDC, rapidly destructive coxopathy.

**Figure 1:**
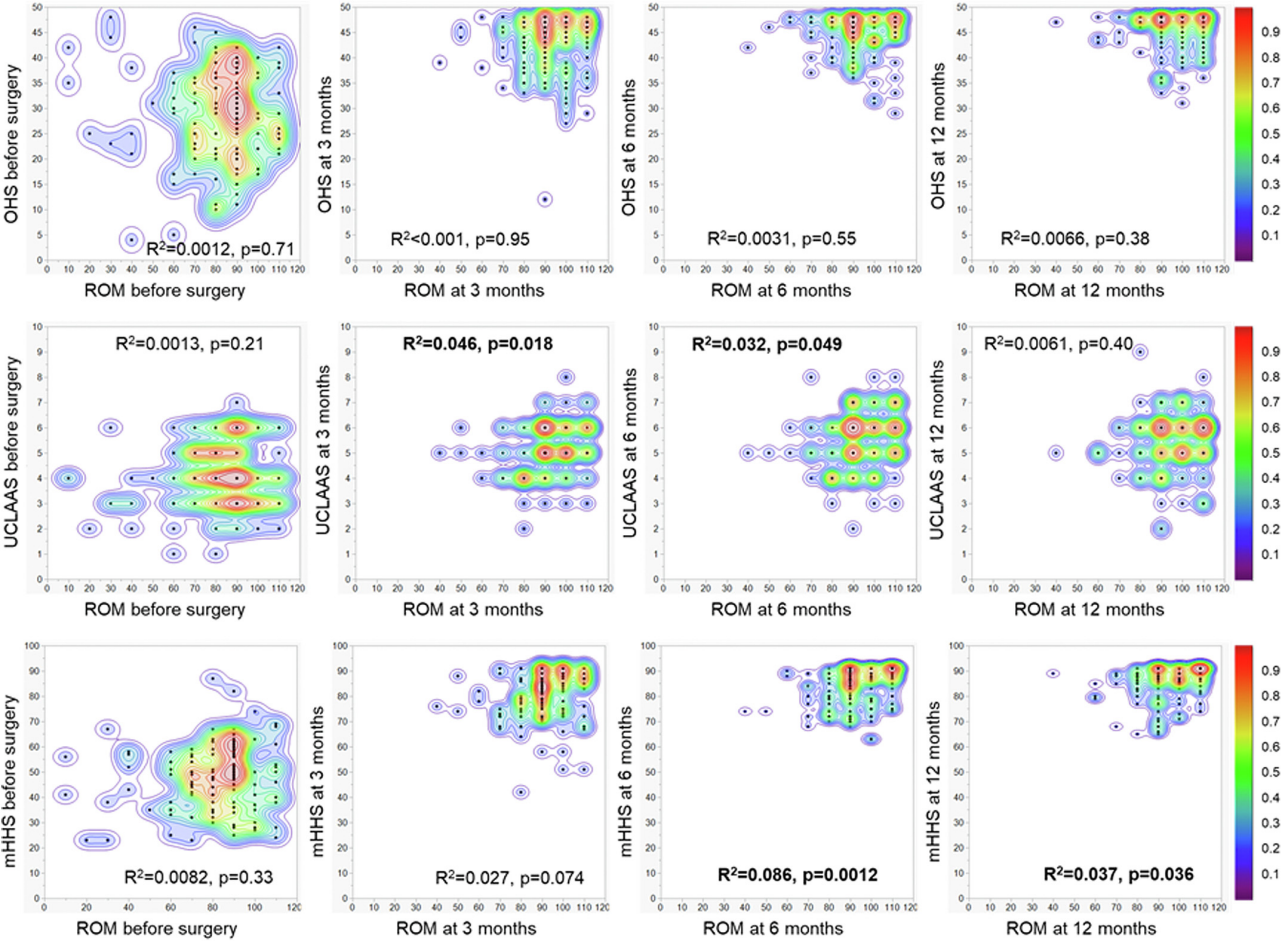
Correlations between flex ROM and each clinical score at each time point (n = 120 for each graph). The distributions were shown as dots and Kernel density estimation.

Correlations between the clinical scores (OHS, UCLAAS, and mHHS) and flexion ROM at each time point until 12 months of surgery are illustrated in Figure 1. The total OHS was not associated with flexion ROM at any time point. UCLAAS was associated with flexion ROM at 3 months (*P* = .018) but not preoperatively or at 6 and 12 months. The total mHHS was significantly associated with flexion ROM at 6 and 12 months (*P* = .012 and *P* = .036, respectively). At 3 months, total mHHS showed a tendency to be higher when flexion ROM was larger, but this correlation was not significant (*P* = .074).

### Comparison between small and large preoperative ROM groups

The total score and scores of each component in OHS, mHHS, and UCLAAS for all patients in the small and large preoperative ROM groups are shown in Table 2. In terms of total scores, there were no significant differences in OHS, mHHS, or UCLAAS between the two groups before surgery and at 3, 6, and 12 months after surgery, although a large ROM group showed a tendency to have a higher total mHHS at 3 (*P* = .07) and 6 (*P* = .063) months. The established value for minimally clinically important difference (MCID) are a difference of 8 points for mHHS [15], 5 points for OHS [16,17], and 0.9 points for UCLAAS [18]. Any of the difference in those three measurements between small and large preoperative ROM groups did not reach the MCID.

**Table 2:** Comparison of each component score between patients with small (<90°) and large (≥90°) preoperative ROM.

			All cases (n = 120)	Small preoperative ROM group (n = 59)	Large preoperative ROM group (n = 61)	*P* value (small group vs large group)
OHS		Pre	28.72 ± 9.39	28.35 ± 10.65	28.95 ± 8.09	0.88
		3 m	42.64 ± 5.87	42.83 ± 5.34	42.49 ± 6.42	0.80
		6 m	44.35 ± 4.16	44.27 ± 3.93	44.62 ± 4.87	0.54
		12 m	44.98 ± 4.00	45.32 ± 3.73	44.67 ± 4.25	0.25
mHHS		Pre	47.55 ± 13.78	46.17 ± 13.56	48.82 ± 13.88	0.29
		3 m	80.4 ± 9.64	78.85 ± 10.08	81.90 ± 9.01	0.070
		6 m	83.88 ± 7.25	82.61 ± 7.54	85.10 ± 6.80	0.063
		12 m	84.90 ± 7.32	84.27 ± 7.60	85.52 ± 7.03	0.27
UCLAAS		Pre	4.07 ± 1.28	3.95 ± 1.27	4.18 ± 1.28	0.50
		3 m	5.15 ± 1.00	4.97 ± 1.00	5.33 ± 0.98	0.074
		6 m	5.51 ± 1.11	5.47 ± 1.16	5.54 ± 1.06	0.76
		12 m	5.42 ± 1.13	5.42 ± 1.22	5.41 ± 1.04	0.75
mHHS	Pain	Pre	18.33 ± 9.59	18.27 ± 8.59	18.39 ± 10.54	0.75
	Pain	12 m	42.54 ± 2.70	42.78 ± 1.86	42.30 ± 3.33	0.90
	**Limp**	**Pre**	**5.43 ± 2.90**	**4.88 ± 3.24**	**5.97 ± 2.44**	**0.045**
	Limp	12 m	10.27 ± 1.56	10.14 ± 1.68	10.40 ± 1.44	0.34
	Support	Pre	7.02 ± 3.12	6.92 ± 3.29	7.11 ± 2.98	0.54
	Support	12 m	9.47 ± 2.56	9.34 ± 2.81	9.60 ± 2.31	0.76
	Distance walked	Pre	6.53 ± 2.76	6.34 ± 2.87	6.72 ± 2.65	0.54
	Distance walked	12 m	9.92 ± 2.06	9.68 ± 2.38	10.15 ± 1.67	0.45
	Stairs	Pre	2.05 ± 0.65	1.97 ± 0.61	2.13 ± 0.67	0.17
	Stairs	12 m	3.08 ± 1.00	2.92 ± 1.00	3.23 ± 0.98	0.083
	**Socks**	**Pre**	**2.37 ± 1.04**	**2.00 ± 0.98**	**2.72 ± 0.97**	**<0.001**
	**Socks**	**12 m**	**3.63 ± 0.82**	**3.42 ± 0.99**	**3.83 ± 0.56**	**0.0071**
	Sitting	Pre	4.85 ± 0.53	4.86 + 0.51	4.84 ± 0.55	0.77
	Sitting	12 m	5.00 ± 0	5.00 ± 0	5.00 ± 0	1.00
	Transport	Pre	0.93 ± 0.25	0.93 ± 0.25	0.93 ± 0.25	0.96
	Transport	12 m	1.00 ± 0	1.00 ± 0	1.00 ± 0	1.00
OHS	Pain	Pre	1.68 ± 1.02	1.85 ± 1.01	1.52 ± 1.01	0.075
	Pain	12 m	3.59 ± 0.69	3.63 ± 0.67	3.56 ± 0.72	0.54
	Washing	Pre	3.11 ± 0.97	3.00 ± 0.96	3.21 ± 0.97	0.18
	Washing	12 m	3.84 ± 0.41	3.83 ± 0.38	3.85 ± 0.44	0.44
	Transport	Pre	2.80 ± 1.06	2.69 ± 1.09	2.90 ± 1.03	0.29
	Transport	12 m	3.78 ± 0.49	3.81 ± 0.47	3.75 ± 0.51	0.41
	Socks	Pre	2.59 ± 1.15	2.37 ± 1.23	2.80 ± 1.03	0.0501
	Socks	12 m	3.78 ± 0.51	3.65 ± 0.56	3.85 ± 0.44	0.054
	Shopping	Pre	3.02 ± 1.26	2.80 ± 1.37	3.23 ± 1.12	0.12
	Shopping	12 m	3.85 ± 0.54	3.92 ± 0.47	3.79 ± 0.61	0.16
	Walking	Pre	2.23 ± 1.14	2.27 ± 1.20	2.18 ± 1.09	0.59
	Walking	12 m	3.62 ± 0.81	3.68 ± 0.80	3.56 ± 0.83	0.20
	Stairs	Pre	2.45 ± 1.10	2.39 ± 1.17	2.51 ± 1.03	0.62
	Stairs	12 m	3.65 ± 0.64	3.73 ± 0.58	3.57 ± 0.69	0.14
	Standing up	Pre	1.22 ± 0.52	2.25 ± 1.31	2.08 ± 1.33	0.46
	Standing up	12 m	3.78 ± 0.52	3.85 + 0.36	3.72 ± 0.64	0.44
	Limp	Pre	1.52 ± 1.48	1.58 ± 1.51	1.46 ± 1.45	0.67
	Limp	12 m	3.67 ± 0.85	3.68 ± 0.92	3.67 ± 0.79	0.58
	Sudden pain	Pre	2.37 ± 1.47	2.42 ± 1.42	2.31 ± 1.52	0.69
	Sudden pain	12 m	3.86 ± 0.47	3.90 ± 0.36	3.82 ± 0.56	0.56
	Work	Pre	2.41 ± 1.23	2.34 ± 1.29	2.48 ± 1.18	0.63
	Work	12 m	3.79 ± 0.58	3.75 ± 0.63	3.84 ± 0.522	0.38
	Night pain	Pre	2.33 ± 1.50	2.39 ± 1.56	2.26 ± 1.44	0.61
	Night pain	12 m	3.78 ± 0.76	3.86 ± 0.57	3.69 ± 0.90	0.24

3 m, 3 months after surgery; 6 m, 6 months after surgery; 12 m, 12 months after surgery; Pre, preoperative.

Bold indicates statistical significance.

Preoperatively, the large preoperative ROM group had higher scores in the mHHS limp (*P* = .045) and socks (*P* < .001) components. At 12 months, there was a significant difference only in the socks component (*P* = .0071).

The large preoperative ROM group had a higher socks component score preoperatively and at 12 months although the difference was not statistically significant (*P* = .0501 and *P* = .054, respectively). No differences were noted between the two groups in other component scores preoperatively or at 12 months.

### Comparison between small and large postoperative ROM groups

The total score and scores of each component in OHS, mHHS, and UCLAAS for both groups are shown in Table 3. There were no significant differences in total OHS, mHHS, and UCLAAS between the groups at 12 months after surgery, although the large postoperative ROM group showed a tendency to have a higher mHHS at 12 months (*P* = .071). Any of the difference in those three measurements between small and large postoperative ROM groups did not reach the MCID.

**Table 3:** Comparison of each component score between patients with small (<90°) and large (≥90°) postoperative ROM at 12 mo.

		Small postoperative ROM (n = 21)	Large postoperative ROM group (n = 99)	*P* value (small group vs large group)
Sex (female)		13 (61.9%)	77 (77.7%)	0.13
Age		61.5 ± 10.1	62.6 ± 12.3	0.53
BMI		24.4 ± 4.2	23.6 ± 3.7	0.33
Diagnosis	OA	21 (100%)	73 (73.7%)	0.030
	ONFH	0	22 (22.2%)	
	RDC	0	4 (4.0%)	
Preoperative flex ROM		56.2 ± 24.6	86.2 ± 15.8	<0.0001
Flex ROM at 12 mo		73.3 ± 10.6	99.6 ± 8.4	<0.0001
OHS total		46.33 ± 2.27	44.7 ± 4.23	0.18
mHHS total		83.43 ± 6.98	85.21 ± 7.38	0.071
UCLAAS		5.43 ± 1.16	5.41 ± 1.12	0.50
Component in HHS	Pain	43.24 ± 1.61	42.39 ± 2.87	0.20
	**Limp**	**9.71 ± 1.79**	**10.39 ± 1.49**	**0.031**
	Support	9.10 ± 2.86	9.55 ± 2.50	0.48
	Distance walked	9.86 ± 2.01	9.93 ± 2.08	0.75
	**Stairs**	**2.57 ± 0.93**	**3.18 ± 0.99**	**0.011**
	**Socks**	**2.95 ± 1.20**	**3.78 ± 0.63**	**<0.0001**
	Sitting	5.00 ± 0	5.00 ± 0	1.00
	Transport	1.00 ± 0	1.00 ± 0	1.00
Component in OHS	Pain	3.81 ± 0.40	3.55 ± 0.73	0.13
	Washing	3.86 ± 0.36	3.84 ± 0.21	0.97
	Transport	3.95 ± 0.22	3.75 ± 0.52	0.076
	Socks	3.81 ± 0.40	3.77 ± 0.53	0.99
	Shopping	4.00 ± 0	3.81 ± 0.60	0.14
	Walking	3.67 ± 0.91	3.61 ± 0.79	0.40
	Stairs	3.81 ± 0.51	3.61 ± 0.67	0.17
	Standing up	3.86 ± 0.36	3.77 ± 0.55	0.63
	Limp	3.81 ± 0.60	3.65 ± 0.90	0.48
	Sudden pain	3.90 ± 0.44	3.85 ± 0.48	0.41
	Work	3.86 ± 0.48	3.78 ± 0.60	0.52
	Night pain	4.00 ± 0	3.73 ± 0.83	0.095

Values are expressed as mean (percent) or mean ± standard deviation.

ONFH, osteonecrosis of the femoral head; RDC, rapidly destructive coxopathy.

Bold indicates statistical significance.

The large ROM group had higher scores in the mHHS limp (*P* = .031), stairs (*P* = .011), and socks (*P* < .0001) components at 12 months.

There was no significant difference in OHS at 12 months between the two groups in any component.

In the socks component at 12 months, the scores for both ROM groups were close (3.81 vs 3.77, *P* = .99).

### The effects of preoperative ROM on clinical measurements

The effects of preoperative flexion ROM on the total score and scores of each component at 12 months after surgery are shown in Table 4. Even after adjusting for sex, age, BMI, and diagnosis (OA or not), a larger preoperative flex ROM was associated with a higher score in mHHS socks component (SC = 0.26, *P* = .0041); however, this effect was not significant on the OHS socks component (*P* = .34).

**Table 4:** Effects of preoperative flex ROM on each component score at 12 mo.

		Unadjusted			Adjusted^a^		
		Standardized coefficient beta	Standard error	*P* value	Standardized coefficient beta	Standard error	*P* value
mHHS 12 m	Total	0.014	0.032	.88	0.036	0.031	.69
Component in HHS	Pain	-0.14	0.012	.13	-0.16	0.012	.083
	Limp	0.073	0.0068	.43	0.070	0.0069	.45
	Support	0.027	0.011	.77	0.077	0.011	.38
	Distance walked	-0.014	0.0090	.88	0.020	0.0090	.83
	Stairs	0.11	0.0044	.23	0.14	0.0044	.13
	**Socks**	**0.26**	**0.0035**	**.0038**	**0.26**	**0.0035**	**.0041**
	Sitting	NA	0	NA	NA	0	NA
	Transport	NA	0	NA	NA	0	NA
OHS	total	-0.040	0.018	.66	-0.033	0.018	.73
Component in OHS	Pain	-0.096	0.0030	.30	-0.097	0.0031	.31
	Washing	-0.081	0.0018	.38	-0.10	0.0018	.28
	Transport	-0.046	0.0021	.62	-0.029	0.0022	.76
	Socks	0.10	0.0028	.27	0.089	0.0028	.34
	Shopping	-0.085	0.0023	.36	-0.071	0.0023	.44
	Walking	-0.0039	0.0036	.97	-0.010	0.0037	.91
	Stairs	-0.00094	0.0028	.99	0.018	0.0029	.85
	Standing up	-0.097	0.0023	.29	-0.087	0.0024	.36
	Limp	0.022	0.0037	.82	0.069	0.0037	.46
	Sudden pain	-0.13	0.0021	.15	-0.15	0.0021	.13
	Work	0.078	0.0025	.40	0.085	0.0026	.37
	Night pain	-0.0080	0.0033	.93	-0.033	0.0034	.73
UCLAAS		-0.045	0.0049	.63	-0.012	0.0049	.89

NA, not applicable.

Bold indicates statistical significance.

a Adjusted for patient factors: age, BMI, sex, and diagnosis (OA or not).

### The effects of postoperative ROM on clinical measurements

The effects of 12-month postoperative flexion ROM on the total score and scores of each component at 12 months after surgery are shown in Table 5. After adjusting for sex, age, BMI, and diagnosis, a larger flexion ROM at 12 months was associated with higher scores in the mHHS support (SC = 0.21 and *P* = .026), stairs (SC = 0.35, *P* = .0002), and socks (SC = 0.32, *P* = .0007) components. At 12 months, flex ROM had no significant effect on any OHS component.

**Table 5:** Effects of flex ROM at 12 mo on each component score at 12 mo.

		Unadjusted			Adjusted∗		
		Standardized coefficient beta	Standard error	*P* value	Standardized coefficient beta	Standard error	*P* value
mHHS 12 m	**Total**	**0.19**	**0.050**	**.036**	0.18	0.051	.054
Component in HHS	Pain	-0.026	0.019	.78	-0.059	0.019	.55
	**Limp**	**0.20**	**0.011**	**.032**	0.14	0.011	.14
	**Support**	**0.18**	**0.018**	**.048**	**0.21**	**0.017**	**.026**
	Distance walked	0.065	0.014	.49	0.064	0.015	.50
	**Stairs**	**0.29**	**0.0066**	**.0014**	**0.35**	**0.0068**	**.0002**
	**Socks**	**0.35**	**0.0054**	**<00,001**	**0.32**	**0.0057**	**.0007**
	Sitting	NA	0	NA	NA	0	NA
	Transport	NA	0	NA	NA	0	NA
OHS	total	-0.038	0.028	.68	-0.044	0.029	.70
Component in OHS	Pain	-0.11	0.0047	.22	-0.12	0.0051	.21
	Washing	-0.0077	0.0028	.93	-0.041	0.0030	.68
	Transport	-0.090	0.0034	.33	-0.085	0.0035	.38
	Socks	0.069	0.0044	.45	0.017	0.0046	.86
	Shopping	0.012	0.0038	.90	0.00097	0.0039	.99
	Walking	0.023	0.0056	.80	-0.0074	0.0060	.94
	Stairs	0.020	0.0044	.83	0.045	0.0046	.64
	Standing up	-0.11	0.0036	.24	-0.13	0.0038	.19
	Limp	-0.026	0.0059	.78	0.052	0.0061	.59
	Sudden pain	-0.020	0.0033	.83	-0.042	0.0035	.67
	Work	0.071	0.0040	.44	0.055	0.0043	.58
	Night pain	-0.079	0.0052	.39	-0.071	0.0055	.46
UCLAAS		0.078	0.0078	.40	0.099	0.0079	.29

NA, not applicable.

Bold indicates statistical significance.

∗ Adjusted for patient factors: age, BMI, sex, and diagnosis (OA or not).

### The distributions of limp, stairs, and socks component scores in mHHS and OHS

**Figure 2:**
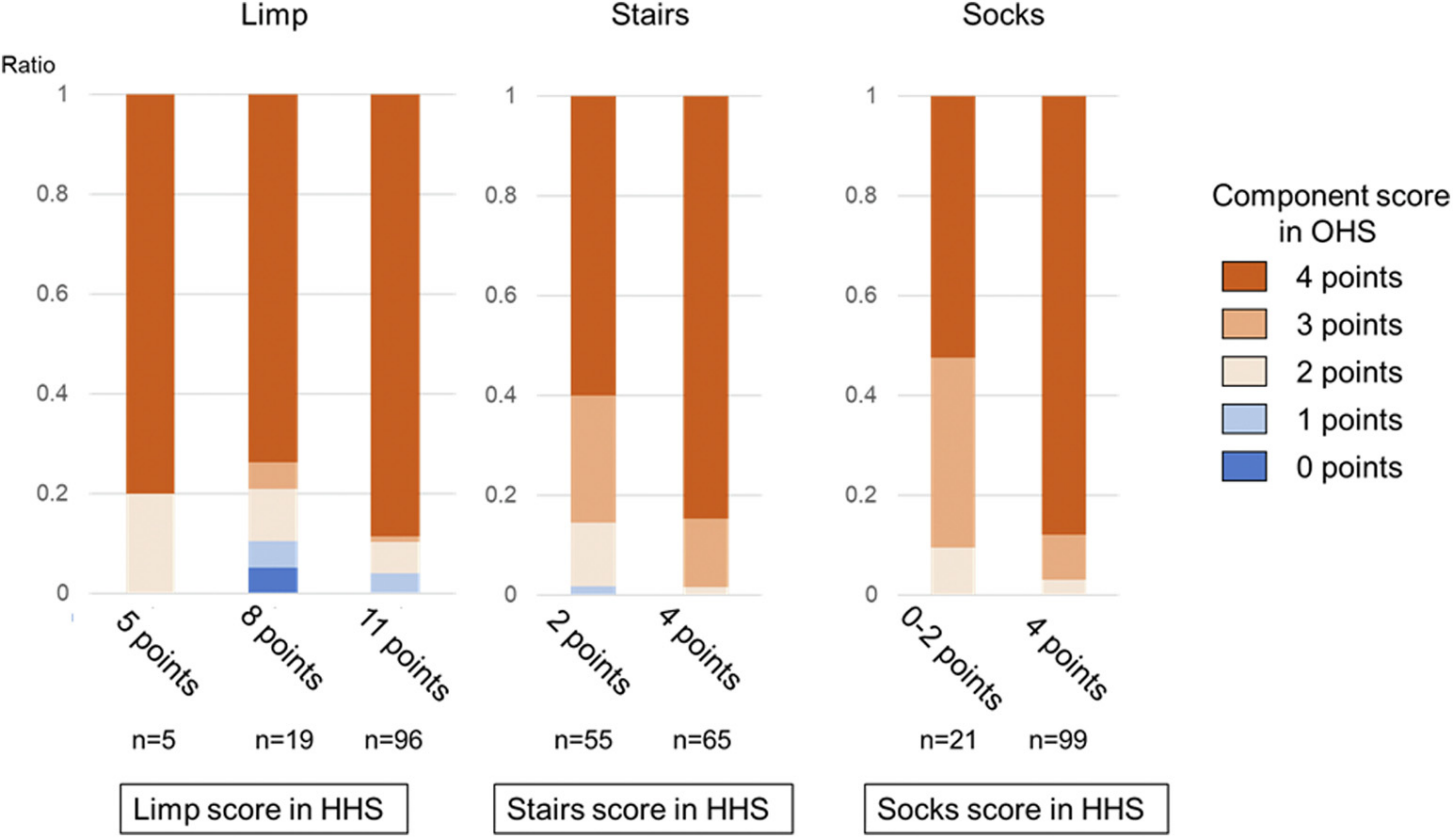
Ratios of each OHS component score for each mHHS component score at 12 months after surgery.

Figure 2 shows the ratios of each response in the OHS questionnaire for each mHHS at 12 months. Even when patients were evaluated by the surgeon as having a low score (5 or 8 out of 11 points) in the mHHS limp component, 75.0% of these patients gave themselves a full score (4 points) in the OHS limp component (Fig. 2). This trend was seen in the stairs and socks components as well; 60% of patients who were evaluated as needing a railing to climb stairs (2 points in the mHHS stairs component) had the full score in the OHS stairs component. Moreover, 52.3% of patients who were evaluated as having difficulty or inability when wearing socks answered that they could wear socks easily (the full score) in the OHS questionnaires.

## Discussion

The mean flexion ROM increased from 80.9° (range: 10°–110°) preoperatively to 95.0° (range: 40°–110°) after 12 months of THA. Despite this improvement, the mean postoperative ROM did not reach normal levels. This may be because the soft tissues have a major impact on impingement-free ROM after THA [19,20]. For most movements, soft-tissue restrictions are considered more important than bony and prosthetic impingements [19]. In the comparison between patients with small (<90°) and large (≥90°) preoperative ROM, none of difference in total mHHS, total OHS, or UCLAAS was statistically significant. None of the difference reached the MCID.

The present study evaluated the effects of preoperative and postoperative flexion ROM on mHHS, OHS, and UCLAAS. Even after adjustments for confounding factors such as age, BMI, sex, and diagnosis, the preoperative ROM was associated only with the postoperative score in the mHHS socks component (SC = 0.26, *P* = .041). However, the postoperative score in the OHS socks component was not affected by preoperative flex ROM.

The 12-month post-THA flex ROM was significantly associated with the mHHS support, stairs, and socks components at 12 months. Interestingly, there were no associations between postoperative flex ROM and any OHS component at 12 months. To our knowledge, no previous studies have discussed the effects of hip ROM on OHS, and this is the first report demonstrating that ROM had no significant effects on OHS.

The effects of flex ROM at each time point on total mHHS and OHS were limited (Fig. 1). There was no significant effect on total OHS at any time point. Although the effects were significant for UCLAAS at 3 and 6 months as well as for total mHHS at 6 and 12 months, the correlation was weak (R^2^ < 0.1).

At 12 months, the effects of ROM were significant in the mHHS support (SC = 0.21, *P* = .026), stairs (SC = 0.35, *P* = .0002), and socks components (SC = 0.32, *P* = .0007), whereas there was no significant effect on any OHS component. However, the difference in component score in mHHS between small and large postoperative groups was small (0 to 0.83; Table 3). Regarding the MCID in each component score in mHHS, there have been no study. Therefore, we cannot conclude that the obtained difference in each component in mHHS was clinically important, even when the difference was statistically significant.

OHS is a PROM questionnaire that patients fill out by themselves, whereas HHS is scored by physicians. OHS and HHS are known to be strongly correlated [9,10]. However, our study found a discrepancy between OHS and HHS in terms of responsiveness to ROM. This may be due to the difference in the evaluation by physicians and patients themselves. THA is an established procedure known to provide high satisfaction rates [21]. As the overall satisfaction is very high after THA, patients may not feel difficulties in daily activities even if physicians deem them as having slight or moderate difficulties. For example, surgeons usually deduct points when patients use a device or take an unusual posture to wear socks, whereas patients may give themselves full score for the socks component even in these situations. In our study, >50% of patients who were deemed by the physicians as having difficulty or inability when wearing socks claimed that they could easily wear socks (the full score) in the OHS questionnaire. Moreover, 75% of patients evaluated as having a low score in the mHHS limp component (5 or 8 out of 11 points) gave themselves a full score in the OHS limp component. Of patients evaluated as needing a railing to climb stairs (2 points in the mHHS stairs component), 60% gave themselves a full score in the OHS stairs component. Thus, patients with limited ROM after THA may not feel as much difficulty in daily activities as surgeons assume. This discrepancy also suggests that the patients have higher satisfaction than that assumed by the surgeons. Surgeons should be aware that a larger postoperative ROM does not necessarily guarantee a higher PROM score, and surgeons may not need to encourage patients to earn a higher flexion ROM.

Kenneth et al. reported that a larger postoperative hip ROM was associated with a higher total mHHS [7]. However, the difference in total mHHS between the smallest and the largest ROM group was 5.7 points, which was still smaller than the established MCID. A higher ROM was associated with a higher score in stairs, limp, and socks components in the study. Our results were consistent with their findings.

To the best of our knowledge, no reports have described the correlation between the UCLAAS and hip ROM. The present study found no correlation between the UCLAAS and postoperative hip flexion ROM at 12 months (Fig. 1 and Table 5). This could be because daily activities do not always require a large hip flexion ROM. For instance, the hip flexion ROM required for cycling is 59.4° [22], whereas that required for activities with deep bending, such as picking up an object while sitting on a chair and squatting on the floor, is 80°–86° [23].

Older age has been associated with a smaller ROM [[24], [25], [26], [27]]. Loss of mobility in older populations might be due to the reduced elasticity of skeletal muscles and ligaments, as well as fat redistribution [28]. Older age is also associated with poorer PROM component scores [29]. To exclude the confounding effects of age, a multivariate model was used to adjust for its effects on function scores. This revealed that a smaller preoperative flex ROM was associated with poorer socks component scores, whereas a smaller postoperative ROM was related to lower support, stairs, and socks component scores.

Quality assessment using PROMs is becoming more important in the field of orthopedics [30,31]. Various PROMs are available for measuring quality after THA [[32], [33], [34], [35]]. Further research is warranted regarding the most responsive PROM to hip ROM.

The limitations of this study include the relatively small number of patients and the heterogeneity in diagnosis and the use of cement. Another limitation is the lack of information on the condition of the lumbar spine. A stiff spine would make various activities, including wearing socks, difficult even with a large hip ROM. The difference in each component score between small and large ROM groups was not compared with MCID because of the lack of established MCID for component score in mHHS and OHS. This would be another limitation for this study.

In conclusion, smaller postoperative flexion ROM was associated with lower scores in the mHHS support, stairs, and socks components after THA even after adjustment for other patient-related confounders. There were no significant associations between ROM and these component scores in the OHS. This discrepancy in responsiveness to ROM between the two measurement tools may be because the HHS uses a surgeon-administered questionnaire, whereas the OHS uses a patient-administered one. The discrepancy also indicates that patients actually have higher satisfaction than that assumed by the surgeons. Surgeons should be aware that patients may not feel as much difficulty because of small ROM as they assume.

## Conflicts of interest

S.M. receives research grant from Kyocera and Smith & Nephew; the other authors declare no potential conflicts of interest.

## Figures and Tables

**Conflict of Interest Statement for Goto:**
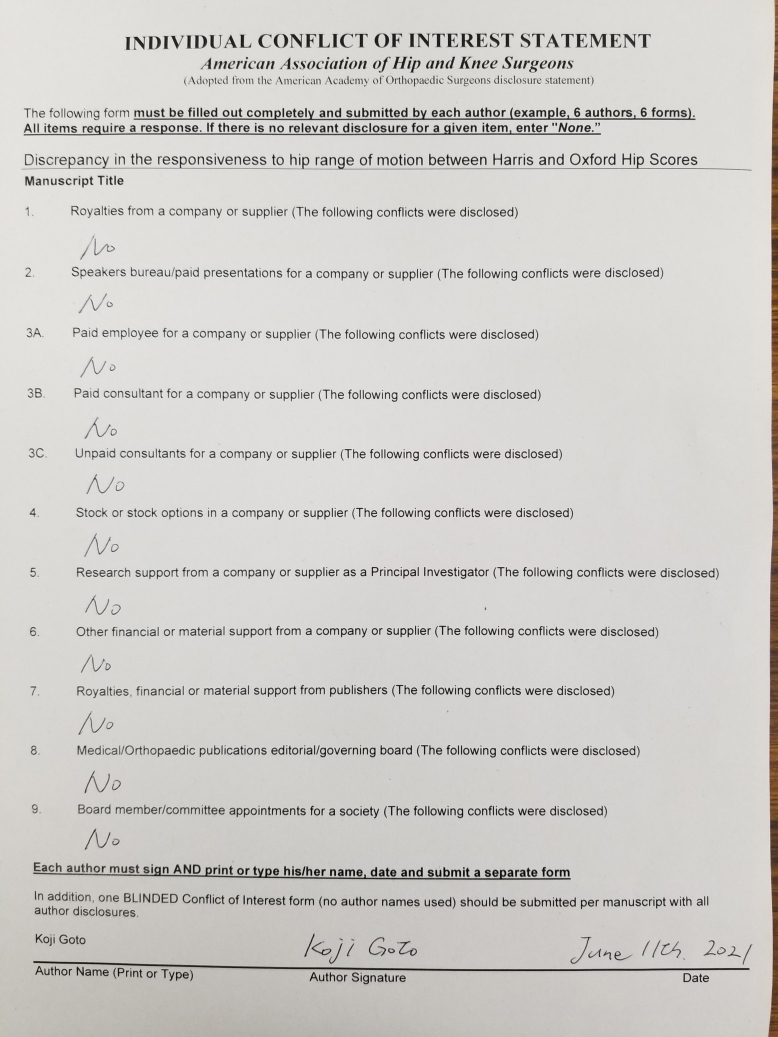


## Supplementary data

Conflict of Interest Statement for Kawai

Conflict of Interest Statement for Kuroda

Conflict of Interest Statement for Okuzu

Conflict of Interest Statement for Matsuda
